# Supplementary material for: Influence of Active and Healthy Ageing on Quality of Life Changes: Insights from the Comparison of Three European Countries
Source: Int J Environ Res Public Health. 2021 Apr 14;18(8):4152. doi: 10.3390/ijerph18084152 (PMC8070976; doi:10.3390/ijerph18084152)
Supplement: Supplementary file 1 [file ijerph-18-04152-s001.pdf]

Supplementary material

Principal component analysis

Multicollinearity problems in the adjusted model were avoided by grouping health, participation and learning-related variables through principal component analysis (PCA). Three PCAs were performed with health, participation and learning-related variables, respectively. The missing PCA variable values were imputed through the Expectation-maximization (EM) algorithm. The component scores were turned into a 0-100 linear scale to make them easier to interpret. The variables were also recoded so that higher component scores indicated better health, greater participation, and higher learning.

The PCA identified three components for the health variables (explained variance, EV: 61.7%-67.8%): good physical and emotional health; functional ability and cognitive and sensory ability. A single component was found for participation (EV: 50.4%-56.7%), and another one for learning (EV: 49.4%-61.9%).

As for the health variables, the good physical and emotional health component included the following variables: number of chronic health conditions obtained from a list of 21 conditions, number of medications, depression (EURO-D **[48]**, where a higher score indicates symptoms of depression) and perceived health status (with 5 Likert-type response categories from "very good" to "very bad"). The functional ability component comprised indicators on limitations to perform a list of 6 basic activities of daily living (ADL) and 9 instrumental activities (IADL), where high values indicate a higher functional impairment. Finally, the cognitive and sensorial ability component grouped the self-perceived memory rating, where a higher score is indicative of cognitive impairment, and presence of eyesight or hearing problems.

The participation component included the number and frequency of participation in the following activities in the last year: voluntary/charity work, sport/social, political/community, read books and magazines, word or number games, play cards or games such as chess.

The learning component comprised indicators regarding the number of years of education, how often they attended courses in the last year, and self-rated reading and writing skills.

48. Prince, M.J.; Reischies, F.; Beekman, A.T.; Fuhrer, R.; Jonker, C.; Kivela, S.L.; Lawlor, B.A.; Lobo, A.; Magnusson, H.; Fichter, M.; et al. Development of the EURO-D Scale--a European, Union Initiative to Compare Symptoms of Depression in 14 European Centres. Br J Psychiatry 1999, 174, 330–338, doi:10.1192/bjp.174.4.330

Table S1. Descriptive statistics of respondents and non-respondents in W6 by country (unweighted)

	Portugal			Spain			Sweden		
	**Participants with complete CASP-12 at W6** **n (row %)**	**Participants with missing CASP-12 at W6** **n (row %)**	**p-value***	**Participants with complete CASP-12 at W6** **n (row %)**	**Participants with missing CASP-12 at W6** **n (row %)**	**p-value***	**Participants with complete CASP-12 at W6** **n (row %)**	**Participants with missing CASP-12 at W6** **n (row %)**	**p-value***
**Age**, mean (SD)	64.0 (8.7)	67.9 (10.8)	<0.001	66.7 (9.6)	71.5 (12.3)	<0.001	69.1 (8.4)	71.8 (10.3)	<0.001
**Sex**			0.537			0.030			0.008
Male	580 (67.3)	283 (32.8)		1062 (64.0)	597 (36.0)		596 (66.4)	303 (33.7)	
Female	753 (69.6)	346 (32.0)		1352 (68.1)	653 (32.9)		764 (71.9)	300 (28.2)	
**Marital status**			<0.001			<0.001			0.639
Without partner	269 (60.7)	175 (39.5)		458 (57.5)	339 (42.5)		328 (70.4)	138 (29.6)	
With partner	1061 (70.8)	454 (30.3)		1864 (69.0)	856 (31.7)		997 (69.3)	443 (30.8)	
**Current job situation**			0.016			<0.001			<0.001
Retired	712 (65.3)	379 (34.7)		919 (61.4)	577 (38.6)		916 (68.3)	425 (31.7)	
Employed	325 (75.2)	117 (27.1)		505 (73.9)	186 (27.2)		403 (76.6)	124 (23.6)	
Homemaker	151 (71.2)	65 (30.7)		710 (71.0)	301 (30.1)		4 (44.4)	5 (55.6)	
Other	125 (68.7)	61 (33.5)		275 (63.5)	159 (36.7)		36 (60.0)	25 (41.7)	
**CASP-12**, mean (SD)	32.3 (4.9)	31.2 (5.7)	<0.001	36.0 (6.2)	34.2 (7.1)	<0.001	39.2 (4.9)	37.4 (6.1)	<0.001
**Physical and emotional health**, mean (SD)	73.7 (12.3)	72.4 (13.8)	0.040	75.7 (12.3)	73.3 (13.8)	<0.001	79.9 (8.1)	76.9 (12.8)	<0.001
**Functional ability**, mean (SD)	76.0 (8.3)	71.9 (15.0)	<0.001	76.6 (8.5)	71.3 (16.4)	<0.001	80.0 (13.2)	76.4 (14.5)	<0.001
**Cognitive and sensorial ability**, mean (SD)	44.0 (15.5)	44.4 (17.8)	0.642	47.4 (15.4)	45.0 (16.9)	<0.001	61.3 (15.1)	56.6 (17.1)	<0.001
**Participation**, mean (SD)	22.1 (17.9)	21.9 (17.8)	0.790	22.6 (18.4)	19.7 (17.2)	<0.001	47.8 (15.6)	43.0 (16.8)	<0.001
**Learning**, mean (SD)	34.3 (18.0)	32.5 (18.5)	0.048	37.8 (16.7)	35.4 (17.2)	<0.001	70.7 (16.1)	65.8 (18.1)	<0.001
**Wealth (€)**, mean (SD)	150.9 (236.9)	130.1 (181.9)	0.153	276.5 (536.8)	315.5 (899.0)	0.236	388.2 (476.5)	253.0 (313.0)	<0.001
